# Duloxetine in the treatment of major depressive disorder: an open-label study

**DOI:** 10.1186/1471-244X-7-43

**Published:** 2007-08-28

**Authors:** James I Hudson, David G Perahia, Inmaculada Gilaberte, Fujun Wang, John G Watkin, Michael J Detke

**Affiliations:** 1 McLean Hospital, Belmont, MA and Department of Psychiatry, Harvard Medical School, Boston, MA, USA; 2 Lilly Research Laboratories, Eli Lilly and Company, Indianapolis, IN, USA; 3 The Gordon Hospital, London, UK; 4 Department of Psychiatry, Indiana University School of Medicine, Indianapolis, IN, USA

## Abstract

**Background:**

Major depressive disorder (MDD) is a chronic and highly disabling condition. Existing pharmacotherapies produce full remission in only 30% to 40% of treated patients. Antidepressants exhibiting dual reuptake inhibition of both serotonin (5-HT) and norepinephrine (NE) may achieve higher rates of remission compared with those acting upon a single neurotransmitter. In this study, the safety and efficacy of duloxetine, a potent dual reuptake inhibitor of 5-HT and NE, were examined.

**Methods:**

Patients (N = 533) meeting DSM-IV criteria for MDD received open-label duloxetine (60 mg once a day [QD]) for 12 weeks during the initial phase of a relapse prevention trial. Patients were required to have a 17-item Hamilton Rating Scale for Depression (HAMD_17_) total score ≥18 and a Clinical Global Impression of Severity (CGI-S) score ≥4 at baseline. Efficacy measures included the HAMD_17 _total score, HAMD_17 _subscales, the CGI-S, the Patient Global Impression of Improvement (PGI-I) scale, Visual Analog Scales (VAS) for pain, and the Symptom Questionnaire, Somatic Subscale (SQ-SS). Quality of life was assessed using the Sheehan Disability Scale (SDS) and the Quality of Life in Depression Scale (QLDS). Safety was evaluated by recording spontaneously-reported treatment-emergent adverse events, changes in vital signs and laboratory analytes, and the Patient Global Impression of Sexual Function (PGI-SF) scale.

**Results:**

The rate of discontinuation due to adverse events was 11.3%. Treatment-emergent adverse events reported by ≥10% duloxetine-treated patients were nausea, headache, dry mouth, somnolence, insomnia, and dizziness. Following 12 weeks of open-label duloxetine therapy, significant improvements were observed in all assessed efficacy and quality of life measures. In assessments of depression severity (HAMD_17_, CGI-S) the magnitude of symptom improvement continued to increase at each study visit, while for painful physical symptoms the onset of improvement was rapid and reached a maximum after 2 to 3 weeks of treatment.

**Conclusion:**

In this open-label phase of a relapse prevention study, duloxetine (60 mg QD) was shown to be safe and effective in the treatment of MDD.

**Trial registration:**

NCT00036309.

## Background

Major depressive disorder (MDD) represents one of the most serious challenges faced by healthcare providers throughout the world, affecting some 18 million people in the United States and 340 million people globally [1]. The negative impact of MDD upon patient well-being and functioning is comparable to that of major chronic medical conditions such as diabetes, hypertension, coronary artery disease, and arthritis [2]. Given the enormous impact of depression upon both the individual patient and the healthcare system as a whole, the need for effective treatment is clear.

Antidepressant medications, in particular the selective serotonin reuptake inhibitors (SSRIs), currently represent the first line of treatment for MDD. SSRIs attained clinical acceptance over tricyclic antidepressants (TCAs) in part due to their improved tolerability profile, including lower rates of anticholinergic events, orthostatic hypotension, sedation, and lower toxicity in overdose [3,4]. However, SSRIs have not demonstrated superior efficacy when compared with TCAs [5], and up to half of all depressed patients fail to respond to SSRI therapy [6]. Thus, the need for alternative safe and effective treatments for MDD is clear.

The antidepressant duloxetine is a potent dual reuptake inhibitor of serotonin (5-HT) and norepinephrine (NE) [7], but lacks significant affinity for muscarinic, histaminergic, α-adrenergic, dopaminergic, serotonergic, and opioid receptors [8]. Previous double-blind, placebo-controlled studies have established the safety and efficacy of duloxetine in the treatment of MDD [9-14]. For licensing purposes, the European Committee for Proprietary Medicinal Products (CPMP) requires that an antidepressant's short-term effect be maintained for a longer duration. For this reason, a randomized withdrawal study, also called a relapse prevention study, was considered the best design. The study included an initial 12-week, open-label treatment phase followed by a 26-week, double-blind, randomized withdrawal phase. The primary results of the relapse prevention study have been published elsewhere [15]. Here we present results from the initial open-label acute phase of a relapse prevention study, in which a cohort of over 500 patients received duloxetine (60 mg once-daily) for 12 weeks.

## Methods

### Study design

This was an open-label study conducted at 29 sites in the United States, France, Italy and Spain. Patients meeting entry criteria received duloxetine 60 mg once-daily for 12 weeks. Study visits were scheduled after 1, 2, 4, 7, 10 and 12 weeks of treatment. Study drug consisted of 2 capsules (30 mg of duloxetine in each capsule) taken once-daily. If necessary due to tolerability, the dose could be reduced to 1 capsule (duloxetine 30 mg/d) at any point during the first 4 weeks of treatment. Patients had to return to 2 capsules once-daily after Week 4 or they were discontinued from the study.

Concomitant medications with primarily central nervous system activity were not allowed, with the exception of benzodiazepines (e.g. lorazepam, diazepam, chlordiazepoxide) and certain hypnotics (chloral hydrate) for a maximum of 6 total days (intermittent or consecutive) during the 12-week period. Use of non-prescription, over-the-counter pain medications (e.g. paracetamol, acetaminophen, ibuprofen) was allowed. Narcotic use was allowed only upon approval by a study physician. Use of certain prescription medications such as ACE inhibitors, antiarrhythmics, or beta-blockers was permitted provided the patient had been on a stable dose for a minimum of 3 months prior to study enrollment.

In accordance with the principles of the Declaration of Helsinki, all patients gave informed consent prior to administration of any study drug or study procedures. The study protocol was approved by both central and local ethical review boards (ERBs) in the 4 countries in which the study was conducted. The participating ERBs included the following: Schulmann and Associates Institutional Review Board (16 sites), Hospital Tarnier Paris-Cochin (4 sites), Policlinico Universitario Udine, Azienda Ospedaliera S. Giovanni Battista, Universita' Di Parma Comitato Etico, Hospital General Yague, Hospital Clinico De Salamanca, Hospital Universitario Nuestra Senora De Valme, Institut Munincipal Assistencia Sanitaria, Hospital General De Vic, and McLean Hospital.

### Selection of patients

All patients met diagnostic criteria for MDD as defined in the *Diagnostic and Statistical Manual of Mental Disorders, 4^th ^Edition *(DSM-IV) [16]. The diagnosis of MDD was confirmed by the Mini International Neuropsychiatric Interview (MINI) [17]. Baseline disease severity was defined by patients' scores on the 17-item Hamilton Rating Scale for Depression (HAMD_17_) [18], and the Clinical Global Impression of Severity (CGI-S) scale [19]. To be eligible for the study, patients were required to have a HAMD_17 _total score ≥ 18 and a CGI-S score ≥ 4 at two consecutive screening visits.

Study participants were adult outpatients at least 18 years of age, and were recruited from several sources including psychiatric health care centers and TV/radio/print advertisements. Patients were excluded for the following reasons: a current and primary Axis I disorder other than MDD, including but not limited to dysthymia; the presence of an Axis II disorder that could interfere with compliance with the study protocol; any previous diagnosis of bipolar disorder, schizophrenia, or other psychotic disorders; any anxiety disorder as a primary diagnosis within the past year; serious suicidal risk; lack of response of the current depressive episode to two or more adequate courses of antidepressant therapy at a clinically appropriate dose for a minimum of 4 weeks, or treatment resistant depression; serious medical illness (including any cardiovascular, hepatic, respiratory, hematologic, endocrinologic, renal, or neurologic disease, or clinically significant laboratory abnormality); initiating or stopping psychotherapy within six weeks prior to enrollment; a DSM-IV-defined history of substance abuse or dependence within the past year; a positive urine drug screen for any substance of abuse.

### Safety assessments

Safety measures recorded at every visit included spontaneously reported treatment-emergent adverse events, supine blood pressure (BP), and heart rate (HR). Elevated blood pressure was defined as supine systolic BP ≥ 140 mm Hg and at least 10 mm Hg greater than baseline, or supine diastolic BP ≥ 90 mm Hg and at least 10 mm Hg greater than baseline. These definitions for elevated blood pressure were based on diagnostic criteria from the Joint National Committee (JNC) on prevention, detection, evaluation, and treatment of high blood pressure [20]. A patient was considered to have a sustained elevation in BP if criteria for elevated systolic or diastolic BP were met at 3 consecutive visits. Potentially clinically significant (PCS) high BP values were defined as: systolic BP ≥ 180 mm Hg and an increase ≥20 mm Hg from baseline, or diastolic BP ≥ 105 mm Hg and an increase ≥15 mm Hg from baseline. A treatment-emergent elevated HR was defined as a value ≥100 bpm with an increase ≥10 bpm from baseline, while a PCS elevated HR was defined as ≥120 bpm and an increase ≥15 bpm from baseline. Criteria used for PCS values for blood pressure and heart rate were developed for previous duloxetine registration studies in conjunction with the FDA's Division of Neuropharmacological Drug Products (DNDP). A PCS value for Fridericia's correction of the QT interval (QTcF) was defined as any postbaseline value ≥450 msec for males or ≥470 msec for females with an increase in QTcF of ≥30 msec from baseline. Blood for hematology was collected at baseline and at the last patient visit (Week 12). Blood for chemistry laboratories was collected at baseline and at Weeks 4 and 12. Normal values for laboratory analytes were based on reference ranges provided by Covance Laboratories, Inc (Princeton, NJ). PCS weight changes were defined as an increase or decrease of ≥7% from baseline body weight.

Changes in sexual functioning were assessed by means of the self-rated Patient Global Impression of Sexual Function (PGI-SF) scale [21], which was collected at baseline and at the last study visit. The PGI-SF is a 4-question instrument that assesses: (i) sexual interest/desire, (ii) erection (for men) or vaginal lubrication (for women), (iii) ability to achieve orgasm, and (iv) an overall rating of sexual function. Each question was rated on a 5-point scale ranging from 1 (no impairment) to 5 (severely impaired).

### Efficacy measures

Efficacy measures included the HAMD_17 _total score; HAMD_17 _subscales, including anxiety/somatization (sum of Items 10, 11, 12, 13, 15, and 17), retardation (sum of Items 1, 7, 8, and 14), sleep (sum of Items 4, 5, and 6), core (sum of Items 1, 2, 3, 7, and 8), and Maier (sum of Items 1, 2, 7, 8, 9, and 10); the CGI-S; the Patient Global Impression of Improvement (PGI-I) scale [19]; Visual Analog Scales (VAS) for pain [22] (six questions regarding the experience of overall pain, headache, back pain, shoulder pain, pain interference with daily activities, and proportion of the day with pain); the Symptom Questionnaire, Somatic Subscale (SQ-SS) [23]; and the pain subscale of the SQ-SS (sum of Items 5 (no pains anywhere), 8 (tight head or neck), 10 (feeling of pressure in head or body), 12 (no aches anywhere), 16 (pressure on head), 19 (muscle pains), 21 (headaches), and 23 (head pains)). Health outcomes were assessed using the Quality of Life in Depression Scale (QLDS) [24] and the Sheehan Disability Scale (SDS) [25].

### Statistical methods

Safety analyses included data from all patients, while efficacy analyses included patients with a baseline and at least 1 postbaseline observation. All results unless otherwise noted were obtained from pre-specified analyses, conducted as described in the statistical analysis plan within the study protocol. The primary outcome of the parent study was an assessment of maintenance of effect of duloxetine 60 mg QD compared with placebo by a comparison of time to relapse among patients who responded to open-label duloxetine treatment. Response was defined as ≥50% decrease in HAMD_17 _total score from baseline, while remission was defined as a HAMD_17 _total score ≤7.

In this single-group, open-label study the changes from baseline to endpoint were analyzed with a paired t-test (compared with zero), using the method of last observation carried forward for subjects who withdrew prematurely. The change from baseline to each visit was analyzed using a mixed-effects model repeated measures (MMRM) approach [26,27] which included only visit and investigator as covariates. Response and remission rates are reported as raw values, and do not control for site variation. For laboratory data, the changes from baseline to endpoint were analyzed using Wilcoxon signed rank test due to non-normality of the changes.

Within this report, the term "significant" is used to denote statistical significance (p ≤ .05).

## Results

### Baseline patient characteristics

A total of 681 patients entered the screening phase of the study, of whom 148 failed to meet entry criteria. Of the 148 patients who did not meet entry criteria, the majority (76%) failed to meet the specific protocol entry criteria (described in the selection of patients section). Other reasons for screen failure included patient decision (17%) and lost to follow-up (4%).

The remaining 533 patients were enrolled into the 12-week open-label acute therapy phase at the 29 study sites (number of patients enrolled at each site: minimum = 2; maximum = 38; median = 17). Baseline patient demographics and psychiatric profiles are presented in Table 1. The median exposure to duloxetine was 81 days, and the study yielded data from approximately 92 patient-years of exposure. Concomitant medications used by ≥5% of patients were ibuprofen, acetaminophen, aspirin, naproxen sodium, and multivitamins. During the first 4 weeks of the study, 80 of 529 patients who started acute therapy at a dose of 60 mg QD (15.1%) required a temporary dose reduction.

**Table 1:** Baseline patient demographics and psychiatric profile

**Characteristic**	**Duloxetine, 60 mg QD (N = 533)**
**Gender, n (%)**	383 (71.9)
Female	
**Age, mean yrs (SD)**	43.4 (12.7)
Age range, yrs	18 – 76
**Weight, mean kg (SD)**	82.1 (22.3)
**Origin, n (%)**	
African descent	34 (6.4)
Caucasian	479 (89.9)
East/Southeast Asian	2 (0.4)
Hispanic	14 (2.6)
Western Asian	1 (0.2)
Other	3 (0.6)
**Psychiatric profile**	
17-Item Hamilton Rating Scale for Depression total score, mean (SD)	23.7 (3.6)
Clinical Global Impression of Severity, mean (SD)	4.55 (0.63)

### Safety

Sixteen patients (3.0%) reported 20 serious adverse events during the 12 week study period. The events occurring in more than 1 patient were suicide attempt (3/533, 0.6%) and suicidal ideation (2/533, 0.4%). One patient died during the course of the study – death was due to suicide. The completed suicide occurred in a middle-aged male after approximately 2 weeks of treatment with duloxetine, and the principal investigator considered the suicide unrelated to the study drug. Other serious adverse events reported in this study were as follows: unstable angina, anxiety, appendicitis, atrial fibrillation, cerebrovascular disorder, chest pain, confusional state, depression, dizziness, feeling abnormal, necrotizing fascitis, perirectal abscess, skin laceration, and vaginal hemorrhage.

A total of 60/533 patients (11.3%) discontinued from the study due to adverse events. The majority of discontinuations (48/60) occurred at the first three study visits – Week 1 (24), Week 2 (7), and Week 4 (17). Adverse events leading to discontinuation in ≥ 0.5% of patients were nausea (11/533, 2.1%), somnolence (4/533, 0.8%), suicide attempt (3/533, 0.6%), and vomiting (3/533, 0.6%). Other reasons for study discontinuation included patient decision (11.6%), loss to follow-up (8.1%), protocol violation (5.1%) and lack of efficacy (1.9%). Treatment-emergent adverse events reported by ≥5% of patients are presented in Table 2. The most commonly reported adverse events were nausea, headache, dry mouth, somnolence, insomnia, and dizziness.

**Table 2:** Treatment-emergent adverse events reported by ≥5% of patients^a^

	**n (%)**
	**Duloxetine, 60 mg QD (N = 533)**

Nausea	191 (35.8)
Headache	108 (20.3)
Dry mouth	96 (18.0)
Somnolence	72 (13.5)
Insomnia	56 (10.5)
Dizziness	54 (10.1)
Diarrhea	53 (9.9)
Constipation	42 (7.9)
Increased sweating	37 (6.9)
Anxiety	33 (6.2)
Decreased appetite	33 (6.2)
Tremor	32 (6.0)
Fatigue	31 (5.8)
Vomiting	28 (5.3)

^a ^Incidence is defined as the percentage of subjects reporting a first occurrence or worsening of the event during the acute phase of the study.

A mean baseline-to-endpoint increase in supine HR of 1.7 bpm was observed during the 12 weeks of therapy (T = 3.9, df = 512, p < .001). Mean changes (SD) in supine systolic and diastolic BP were an increase of 1.4 (12.8) mm Hg and 0.7 (9.3) mm Hg, respectively (T = 2.4, df = 512, p = .017, and T = 1.7, df = 512, p = .084, respectively). The incidence of treatment-emergent sustained elevations in blood pressure were: systolic BP – 5/513 (1.0%); diastolic BP – 4/513 (0.8%); either systolic or diastolic BP – 8/513 (1.6%). No patients had PCS low values for blood pressure or heart rate. The incidence of PCS high values were: systolic BP – 1/513 (0.2%); diastolic BP – 4/513 (0.8%); heart rate – 1/513 (0.2%).

Patients had a mean decrease in body weight of -0.1 kg [SD = 2.4] (T = -0.7, df = 428, p = .50). One patient (0.2%) experienced a PCS weight loss, while two patients (0.5%) had a PCS weight gain.

Of the 225 patients having an ECG classified as normal at baseline and having at least 1 post-baseline measurement, 12.4% (28/225) had a treatment-emergent abnormal ECG. No patients (0/402) had a treatment-emergent PCS QTcF interval.

Baseline-to-endpoint mean changes on all 4 items of the PGI-SF were negative, indicating an improvement in sexual functioning. When analyzed separately by gender (Table 3), female patients demonstrated significantly greater improvement compared with male patients on item 2 (erection/vaginal lubrication), item 3 (ability to achieve orgasm), and item 4 (overall sexual functioning). The incidence of spontaneously-reported adverse events related to sexual functioning was: erectile dysfunction 2.4%, delayed ejaculation 2.3%, decreased libido 2.3%, anorgasmia 1.7%, abnormal orgasm 1.1%.

**Table 3:** PGI-SF – mean change by gender

	**Gender**	**Baseline, mean (SD)**	**Change, mean (SD)**	**p-value**^a^	**Between-gender p-value**^b^
**Item 1 Sexual interest/desire**	Female (n = 280)	3.57 (1.53)	-1.01 (1.62)	T = -10.5, df = 279, p < .001	F(1, 370) = 0.9, p = .350
	Male (n = 119)	3.25 (1.44)	-0.71 (1.67)	T = -4.7, df = 118, p < .001	
**Item 2 Erection (male); vaginal lubrication (female)**	Female (n = 270)	2.46 (1.56)	-0.53 (1.52)	T = -5.8, df = 269, p < .001	F(1, 360) = 7.6, p = .006
	Male (n = 119)	2.72 (1.46)	-0.29 (1.66)	T = -1.9, df = 118, p = .055	
**Item 3 Ability to achieve orgasm**	Female (n = 267)	3.25 (1.64)	-0.74 (1.74)	T = -7.0, df = 266, p < .001	F(1, 354) = 4.3, p = .038
	Male (n = 116)	2.55 (1.48)	0.14 (1.74)	T = 0.9, df = 115, p = .396	
**Item 4 Overall sexual functioning**	Female (n = 269)	3.40 (1.59)	-0.87 (1.64)	T = -8.7, df = 268, p < .001	F(1, 359) = 4.9, p = .027
	Male (n = 119)	2.96 (1.50)	-0.27 (1.71)	T = -1.7, df = 118, p = .089	

^a ^within-stratum p-values from paired t-test.

^b ^p-values for gender from ANCOVA with baseline PGI-SF, investigator, and gender in the model.

PGI-SF = Patient Global Impression of Sexual Functioning

Statistically significant mean baseline-to-endpoint changes were observed in some laboratory analytes (e.g. gamma glutamyltransferase: mean change -0.69 U/L (SD = 23.8), Wilcoxon p = .002; alkaline phosphatase: mean change 1.46 U/L (SD = 10.9), Wilcoxon p = .006). However, the incidence of treatment-emergent abnormal laboratory values was low. The only analyte with an incidence of abnormal values ≥10% was high creatine phosphokinase (11.4%). In the case of liver enzymes, the incidence of alanine transaminase (ALT) and aspartate transaminase (AST) values greater than or equal to 1, 3, 5, 10, and 20 times the upper limit of normal (ULN) were *a priori *specified analyses. During acute-phase treatment, 37/377 patients (9.8%) had ALT values that were greater than 1×, but less than 3×, the ULN. One patient (0.2%) had an ALT elevation greater than 3×, but less than 5×, the ULN, while no patients had ALT values that were ≥5× ULN. In the case of AST, 37/393 patients (9.4%) had a reading that exceeded 1×, but did not exceed 3×, the ULN. Two patients (0.5%) had an AST elevation greater than 3×, but less than 5×, the ULN, and 1 patient (0.2%) experienced an AST elevation greater than 5×, but less than 10×, the ULN. In the 28 patients with normal or low bilirubin at baseline who then had treatment-emergent abnormal ALT during acute phase therapy, 1 patient (3.6%) had a corresponding treatment-emergent abnormal high bilirubin value. The mean baseline-to-endpoint change in total bilirubin did not achieve statistical significance (0.07 μmol/L (SD = 3.2), Wilcoxon p = .584), while the incidence of abnormal high total bilirubin at anytime was 0.7%. While statistically significant within-group mean changes were observed for some laboratory values, the magnitude of these changes was not considered clinically relevant in light of the small number of treatment-emergent abnormal values. Abnormal values in hepatic enzymes were usually transient and of low magnitude. Increases in ALT/AST values were associated with increases in bilirubin in 1 (3.6%) patient, but the finding was not considered clinically relevant.

### Efficacy

**Figure 1:**
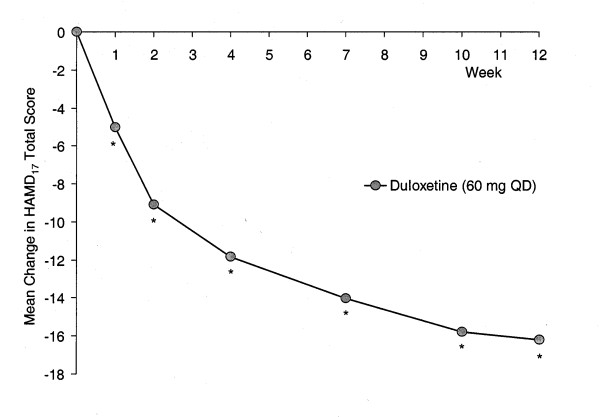
Visitwise plot of mean change in HAMD_17 _total score for patients receiving duloxetine (60 mg QD, n = 511). MMRM analysis. * p < .001 from t-test for LS mean change = 0.

All assessed depression efficacy measures (HAMD_17_, CGI-S, PGI-I) showed significant improvement following 12 weeks of duloxetine therapy (Table 4). A visitwise plot of mean change in HAMD_17 _total score for patients receiving duloxetine (60 mg QD) is presented in Figure 1.

**Table 4:** Summary of efficacy measures

	**Baseline, mean (SD)**	**Endpoint, mean (SD)**	**p-value**^a^
**Hamilton Rating Scale for Depression total score **(n = 511)	23.7 (3.6)	9.9 (7.7)	T = -39.1, df = 510, p < .001
**Hamilton Rating Scale for Depression subscales **(n = 511)			
Core	9.5 (1.7)	3.4 (3.7)	T = -37.2, df = 510, p < .001
Maier	12.2 (2.0)	4.5 (4.4)	T = -38.1, df = 510, p < .001
Anxiety/Somatization	7.6 (1.9)	3.6 (2.7)	T = -30.7, df = 510, p < .001
Retardation	8.2 (1.5)	3.4 (3.1)	T = -35.3, df = 510, p < .001
Sleep	3.8 (1.7)	1.7 (1.8)	T = -23.5, df = 510, p < .001
**Clinical Global Impression of Severity **(n = 512)	4.6 (0.6)	2.3 (1.3)	T = -36.1, df = 511, p < .001
**Patient Global Impression of Improvement **(n = 511)	N/A	2.6 (1.4)	N/A

LOCF mean change analysis

^a ^p-values are from paired t-test for mean change from baseline to endpoint = 0.

The rate of response at Week 12 was 67.9% (347/511 patients), while the endpoint remission rate was 52.8% (270/511 patients; LOCF analyses). Utilizing the entry criteria for the relapse-prevention phase of this study (no longer meeting DSM-IV criteria for MDD; HAMD_17 _total score ≤9; CGI-S score ≤2), 280/533 patients (52.5%) met the entry criteria.

Analyses of mean change in HAMD_17 _total score revealed no significant treatment-by-strata interactions for age (<55 years vs. ≥55 years), gender, ethnic origin (Caucasian vs. other), baseline HAMD_17 _score (<19 vs. ≥19, or <25 vs. ≥25), number of previous major depressive episodes (<median vs. ≥median), or treatment history (no previous antidepressants vs. ≥1 previous antidepressant therapy).

**Figure 2:**
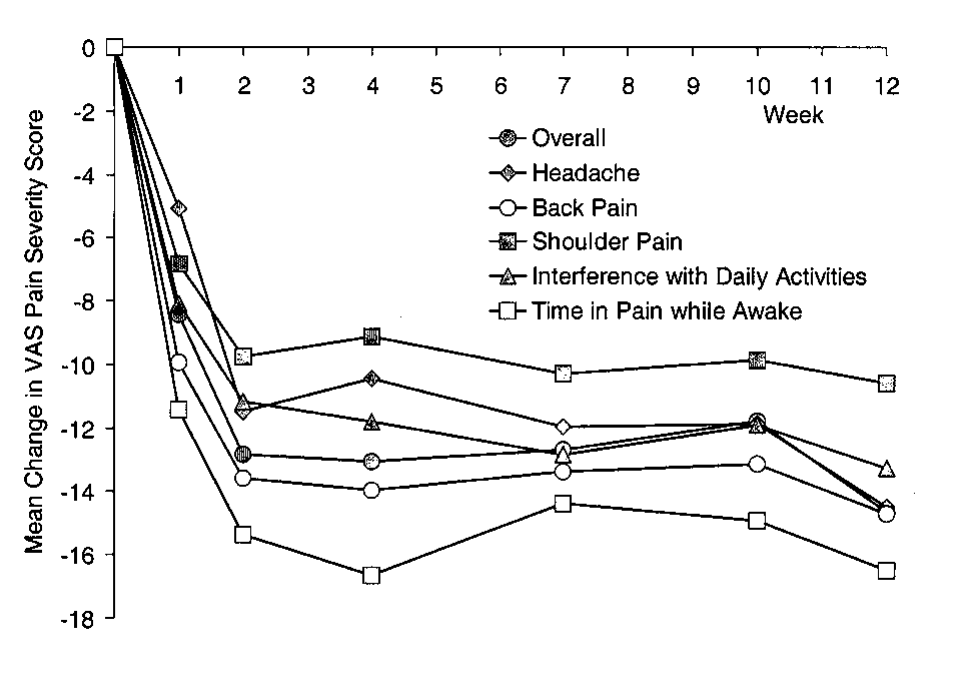
Visitwise plot of mean change in VAS pain severity scores for patients receiving duloxetine (60 mg QD). MMRM analysis. p < .001 for each item at all visits (t-test for LS mean change = 0).

Scales assessing the severity of painful physical symptoms (VAS) and somatic symptoms (SQ-SS) showed significant improvement following 12 weeks of duloxetine therapy (Table 5). A visitwise plot of mean changes in the six assessed VAS pain severity questions is shown in Figure 2. Both assessments of quality of life (SDS, QLDS) also demonstrated significant improvement from baseline to endpoint (Table 6).

**Table 5:** Summary of physical symptom efficacy measures

	**Baseline, mean (SD)**	**Endpoint, mean (SD)**	**p-value**^a^
**VAS pain severity**			
Overall (n = 504)	33.8 (26.6)	21.3 (25.6)	T = -10.5, df = 503 p < .001,
Headache (n = 504)	27.6 (27.8)	15.3 (22.4)	T = -9.4, df = 503, p < .001
Back pain (n = 504)	28.7 (29.3)	15.8 (23.3)	T = -10.7, df = 503, p < .001
Shoulder pain (n = 501)	23.1 (28.9)	14.1 (23.2)	T = -7.7, df = 500, p < .001
Interference with daily activities (n = 501)	28.2 (28.2)	17.3 (25.3)	T = -8.8, df = 500, p < .001
Time in pain while awake (n = 500)	38.3 (32.2)	23.3 (28.6)	T = -10.3, df = 499, p < .001
**Symptom Questionnaire, Somatic Subscale **(n = 492)	12.4 (5.3)	7.6 (5.1)	T = -19.5, df = 491, p < .001
**Symptom Questionnaire, Somatic Subscale pain items **(n = 504)	4.9 (2.4)	3.5 (2.6)	T = -11.9, df = 503, p < .001

LOCF mean change analysis

^a^p-values are from paired t-test for mean change from baseline to endpoint = 0

VAS = Visual Analog Scale

**Table 6:** Summary of health outcome measures

	**Baseline, mean (SD)**	**Endpoint, mean (SD)**	**p-value**^a^
**Quality of Life in Depression Scale **(n = 385)	19.0 (7.7)	8.2 (8.7)	T = -22.6, df = 384, p < .001
**Sheehan Disability Scale**			
Total (n = 419)	18.7 (5.6)	9.5 (7.8)	T = -22.9, df = 418, p < .001
Work (n = 315)	5.7 (2.5)	3.0 (2.8)	T = -15.6, df = 314, p < .001
Social life (n = 419)	6.6 (2.3)	3.3 (2.9)	T = -22.1, df = 418, p < .001
Family life (n = 420)	6.3 (2.3)	3.2 (2.8)	T = -19.5, df = 419, p < .001

LOCF mean change analysis

^a^p-values are from paired t-test for mean change from baseline to endpoint = 0

## Discussion

The results of the current investigation are based on the initial 12-week, open-label acute treatment phase of a randomized withdrawal study of duloxetine in the prevention of relapse of MDD. Results of the parent study have been published and show that patients receiving duloxetine 60 mg/d had significantly longer times to relapse than patients receiving placebo [15]. The current analysis utilized data from an open-label setting to assess the safety and efficacy of a once-daily 60 mg duloxetine dose during acute phase treatment of MDD. Although the study yielded data from a cohort of over 500 patients receiving duloxetine at the target therapeutic dose for up to 12 weeks, interpretation of results from an open-label study should be approached cautiously. The absence of placebo or active comparator treatment arms limits our ability to draw firm conclusions from the current results, especially with regard to efficacy outcomes. Patients in an open-label study will exhibit the combined benefits of both drug and placebo responses, and therefore efficacy results such as response and remission rates may be more favorable than those obtained from double-blind, placebo-controlled studies. Furthermore, the statistical significance of treatment outcomes can only be assessed relative to baseline values rather than a comparator group. However, an open-label setting does provide a more realistic approximation of clinical practice when compared with a double-blind study, and a large study such as this can yield a substantial body of safety and tolerability data. In certain areas, such as rates of discontinuation and the incidence of spontaneously-reported adverse events, results from an open-label study may be especially relevant for practicing clinicians. With these limitations in mind, we discuss in general terms the principal findings from this study. Wherever possible, comparisons with data from previous double-blind, placebo-controlled studies are utilized to provide context for the current results.

The rate of discontinuation due to adverse events in this study (11.3%) was similar to that observed in double-blind, placebo-controlled studies of duloxetine, 60 mg once-daily (13.8% [9] and 12.5% [10]) and is also broadly consistent with the reported rates of discontinuation due to adverse events presented in the full prescribing information for other antidepressants (6% escitalopram [28], 10% paroxetine controlled release [29], 11% venlafaxine extended release [30]). Furthermore, the incidence and pattern of treatment-emergent adverse events was also consistent with previously published data. In an analysis of pooled data from placebo-controlled studies of duloxetine (40–120 mg/d), adverse events occurring in ≥5% of duloxetine-treated patients, and at twice the rate for placebo, were nausea, dry mouth, fatigue, dizziness, constipation, somnolence, decreased appetite, and increased sweating [14]. The incidence of treatment-emergent nausea in the present study (35.8%) was similar to that reported in 2 placebo-controlled studies of duloxetine 60 mg QD [9,10]. Although patients could request a reduction of the duloxetine dose from 60 mg QD to 30 mg QD at any point during the first 4 weeks of therapy, 85% of patients tolerated the starting dose of 60 mg QD without the need for downward titration.

The incidence of spontaneously-reported adverse events related to sexual functioning was relatively low. However, spontaneous reports frequently under-represent the actual incidence of sexual side-effects, and more accurate estimates may be elicited using validated, structured questionnaires. In the present study, the Patient Global Impression of Sexual Function (PGI-SF) questionnaire was utilized to assess treatment-emergent impairment of sexual functioning. Female patients experienced significant within-group mean improvements on all 4 items of the PGI-SF, while male patients experienced improvement on Item 1 (sexual interest/desire). While results from an open-label study should be viewed with a degree of caution, these results suggest that duloxetine does not have a substantial adverse impact upon patient's perception of sexual functioning. By way of comparison, in four double-blind, placebo- and paroxetine-controlled studies of up to 9 months duration, the Arizona Sexual Experience (ASEX) scale was utilized to compare the incidence of sexual dysfunction in patients receiving duloxetine with the corresponding rates in paroxetine- and placebo-treated patients. Results of an analysis of pooled data from these studies showed that the incidence of acute-phase sexual dysfunction in patients receiving duloxetine (40–120 mg/d) was significantly lower than that for patients receiving paroxetine (20 mg QD) [31].

Consistent with duloxetine's pharmacological profile as a reuptake inhibitor of NE, a mean increase in heart rate was observed (1.7 bpm from baseline to endpoint). The magnitude of this increase is similar to that observed in an analysis of pooled data from acute-phase, placebo-controlled studies of duloxetine (1.4 bpm vs. -0.6 bpm for placebo) [32]. Modest increases in HR have also been observed during treatment of depressed patients with the NE reuptake inhibitor reboxetine (increase in HR of approximately 8% during 3 weeks of treatment at 4–8 mg/d) [33] and the 5-HT and NE reuptake inhibitor venlafaxine (increase of 3.8 bpm during 8 weeks of treatment at 225 mg/d) [34]. Duloxetine also produced small (less than 2 mm Hg) mean increases in blood pressure – in the case of supine systolic BP the mean change to endpoint (1.4 mm Hg) was statistically but not clinically significant when compared with the baseline value. These mean changes are consistent with those observed in acute-phase, placebo controlled studies of duloxetine (40–120 mg/d) [32]. Thus, in an analysis of pooled data, mean change in supine systolic BP was 0.8 mm Hg for duloxetine vs. -1.4 mm Hg for placebo (p < .001), while mean change in supine diastolic BP was 0.9 mm Hg for duloxetine vs. 0.4 mm Hg for placebo (p = .099) [32].

In the present study, duloxetine lacked significant effects on the QT interval. None of the 402 patients providing ECG data had a PCS prolongation of corrected QT interval. These data are consistent with results obtained from placebo-controlled studies of duloxetine, which found no evidence for prolongation of QTc intervals in duloxetine-treated patients when compared with those receiving placebo [32].

During the course of this 12-week study, patients receiving duloxetine had a small (0.1 kg) decrease in mean body weight. In the absence of a placebo comparator, no definitive conclusions can be drawn concerning the significance of this mean change. However, in an analysis of pooled data from acute-phase, placebo-controlled trials of up to 12 weeks duration, patients receiving duloxetine (40–120 mg/d) exhibited a mean change in weight of -0.46 kg, compared with 0.23 kg for those receiving placebo (p < .001) [32]. Furthermore, results from a long-term (52-week) open-label study of duloxetine (80–120 mg/d) demonstrated that mean body weight decreased slightly in the first few weeks of treatment, returned to baseline levels at intermediate visits, and showed an increase of 1.1 kg at the study endpoint [35].

In a large study with many measures, small clinically insignificant mean changes in laboratory values commonly achieve statistical significance. Thus, in this large study, mean changes in some laboratory values were statistically significant, but small in magnitude and of doubtful clinical relevance. However, definitive evidence of non-causality is problematic in the absence of placebo control. In the present study, the incidence of ALT and AST values greater than 1×, but less than 3× the ULN, were 9.8% and 9.4%, respectively, while 1 patient (0.2%) had an ALT elevation greater than 3× the ULN. These results are consistent with observations from controlled studies of duloxetine. Within the primary placebo-controlled database (pooled data from 8 studies), the incidence of abnormal (high) values for ALT (duloxetine 9.5% vs. placebo 7.4%; p = .146) and AST (duloxetine 8.1% vs. placebo 6.0%; p = .122) did not differ significantly from the placebo rate [32,36]), while elevations of ALT greater than 3× the ULN occurred in 0.9% (8/930) of duloxetine-treated patients compared with 0.3% (2/652) of those receiving placebo [36]. Analyses from another long-term (52-week), open-label clinical study of duloxetine (N = 2109) showed that duloxetine use was associated with a mild, transient, self-limited rise in ALT and AST, and these changes did not appear to be of clinical significance [35,36].

The efficacy of duloxetine (40–120 mg/d) in the acute treatment of MDD has been established in a number of acute phase, double-blind, placebo-controlled studies, in addition to a long-term open-label study [9-14,35]. Efficacy results obtained in this study are consistent with those obtained previously at a 60 mg once daily dose [9,10], although double-blind, placebo-controlled studies should be regarded as the primary source of efficacy data, with open-label studies such as this playing a supporting role. The time course of improvement in individual symptom domains is noteworthy. In assessments of depression severity (HAMD_17_, CGI-S) the magnitude of improvement continued to increase at each study visit, while for painful physical symptoms the onset of improvement was rapid and reached a maximum after 2 to 3 weeks of treatment. With regard to duloxetine's effect on overall pain severity improvement, our observations are consistent with experimental data indicating that both 5-HT and NE exert analgesic effects *via *descending pain pathways [37-39]. The effects on painful symptoms observed in this study are consistent with data from other duloxetine studies demonstrating analgesic effects in depressed patients [40], and the results from the current study are also supportive of those obtained previously from double-blind, placebo-controlled studies which suggest that duloxetine's effect upon painful physical symptoms is, to some extent, independent of its effects upon core emotional symptoms of depression. Thus, in a path analysis of pooled data from 2 placebo-controlled clinical trials, 50% of duloxetine's total effect on overall pain severity was found to be independent of changes in depressive symptom severity [41].

In the current study, the rate of treatment response (≥50% reduction in HAMD_17 _total score from baseline) was 67.9%, while the remission rate (HAMD_17 _total score ≤7) was 52.8% (LOCF analysis). In two previously-published placebo-controlled, 9-week studies of duloxetine (60 mg once-daily), estimated probabilities of response (MMRM analysis) were 62% [9] and 65% [10] while probabilities of remission were 44% [9] and 43% [10]. In other placebo-controlled studies of duloxetine at doses up to 120 mg/d probabilities of remission (MMRM) of up to 57% have been observed [14]. The remission rate in the present study is somewhat higher than that observed in the two placebo-controlled trials involving duloxetine 60 mg once-daily dosing [9,10]. As discussed previously, this may be a result of the more favorable treatment outcomes often observed in patients participating in an open-label study. However, other confounding factors also preclude any detailed between-study comparisons, including differing treatment periods (12 weeks in this study vs. 9 weeks in the placebo-controlled studies), somewhat higher baseline severity of depression in the present study (HAMD total score of 23.7 vs. 20–21 in the placebo-controlled studies), and differing analytical methods (LOCF in the current study vs. MMRM in the placebo-controlled studies).

In summary, both safety and efficacy results from this open-label study are supportive of those obtained from more rigorous double-blind, placebo-controlled trials. The cohort of 533 patients in this study was the largest patient group to receive the recommended therapeutic dose of 60 mg once daily in any duloxetine study to date. Despite the limited utility of efficacy data obtained under open-label conditions, it is hoped that the results described here will provide clinically relevant information for practicing clinicians, especially with regard to the safety and tolerability of duloxetine.

A number of study limitations should be considered when interpreting the present results. As mentioned previously, the principal limitation is the open-label nature of the study and the lack of placebo or active comparator treatment arms. Secondly, the study was of 12 weeks duration and therefore the results are applicable only to acute phase treatment of MDD. Thirdly, patients received a fixed 60 mg dose of duloxetine throughout the study, although the option of a temporary downward titration to 30 mg QD was available at the beginning of the treatment period. Outcomes may have differed if dosing regimens had been optimized on an individual patient basis. Fourthly, patients with serious or unstable medical illnesses were excluded from the study, which limits the generalizability of the current results to a general population of depressed patients.

## Conclusion

In this open-label study of over 500 patients with a diagnosis of MDD, duloxetine (60 mg QD) was safe and efficacious in the treatment of both emotional and painful physical symptoms of depression. Safety and tolerability results were consistent with those observed previously in double-blind, placebo-controlled studies.

## Competing interests

DP, IG, FW, JW, and MD were employees of Eli Lilly and Company at the time this manuscript was drafted. DP, IG, FW, and MD own Eli Lilly and Company stock. JH has consulted for Eli Lilly and Company and Ortho-McNeil Pharmaceutical, Inc.; and has received research grants from Eli Lilly and Company, Ortho-McNeil Pharmaceutical, Inc., and Forest Laboratories. The article-processing charge for this manuscript is being paid by Eli Lilly and Company.

## Authors' contributions

DP, IG, and MD conceived of the study, participated in its design and coordination, interpreted the data, and helped to draft the manuscript. FW performed the statistical analysis and interpreted the data. JH participated as a site principal investigator in the study and helped interpret the data. JW interpreted the data and drafted the manuscript. All authors critically reviewed and approved the final manuscript.

## Pre-publication history

The pre-publication history for this paper can be accessed here:


